# A Case of Lupus Exedens of Face

**Published:** 1885-09

**Authors:** T. C. Osborn

**Affiliations:** Honorary Member Texas State Medical Association, Cleburne, Texas, Cleburne, Texas


### Medical ★ Journal,

TEXAS

PUBLISHED MONTHLY AT

AUSTIN, TEXAS.

Vol. 1.] SEPTEMBER, 1885. [No. 3.

*"Scribimus indocti, doctique!"*

### ORIGINAL ARTICLES.

☞ CONTRIBUTED EXCLUSIVELY TO THIS JOURNAL. ☜

*The Articles in this Department are accepted, and published with the understanding that we are not responsible for, nor do we indorse the views and opinions of the writers, by so doing.*

### A CASE OF LUPUS EXEDENS, OR EPITHELIOMA OF THE FACE.

*By T. C. Osborn, M. D., Honorary Member Texas State Medical Association, Cleburne, Texas.*

Read before Johnson County Medical Society, and by Society resolution contributed to Daniel's Texas Medical Journal.

THERE came to me for treatment on the 1st of March, 1874, Miss Fannie Tarbutton, aged 13 years, fair complexion light hair and blue eyes, a sallow and badly grown girl, from Delhi, Louisiana.

She was accompanied by her father and step-mother, who gave me a complete history of the case, and urged me to receive her as a house patient, adding their honest convictions that she could hardly survive the summer season, unless I could give her a better course of treatment than she had been subjected to during the past three years, by other physicians.

Owing to the frightful ravages of the disease, and the extremely offensive odor of the ulcerated surface, I hesitated about adopting her as a patient in my family, until my youngest daughter, who was of the same age, solicited so strongly for the sufferer, urging that she would act in the capacity of her nurse, and carry out my directions implicitly, if I would let her remain in her care. To oblige my daughter, whose sympathies were so strongly elicited in favor of the poor little sufferer, I reluctantly consented to accept the patient, with the understanding that I would do everything in my power for her relief, if not the cure of the repulsive disease. I had in the meantime become convinced that the parents were only too willing to put the child as far away from themselves as possible, under the plea of my skill, as the offensive odor was too disagreeable for the recently initiated olfactorics of the sensative step-mother to allow the haunting presence of the afflicted girl to remain any longer under her own inspection.

The patient was a native of Georgia, but had resided in the paludal regions of Louisiana for seven years, during which time she had repeatedly suffered from protracted spells of intermittent fever, and, in the summer of 1871, after an unusually severe attack, which left her utterly prostrated, she was seized with an eczematous eruption of the face and eyes, which terminated in a pimple of lupus on the left side of the nose, which extended rapidly over the nose, cheeks and upper lip, denuding the surface frightfully in its march, and creating almost insupportable pain, and profuse suppuration. And, as she was the cook and washerwoman of the family, it can be easily conceived, that the disease was not likely to be arrested as long as she remained at home. The whole face was much swollen during these many months, and at the time of coming to me the nose and upper lip had sloughed away entirely, leaving only the exposed nasal apertures for breathing purposes, and the bare gum and teeth for mastication. One of the front teeth had been removed on account of its looseness, and several other upper teeth were quite loose in the sockets from extension of the ulceration along the border of the gum. Through the opening made by the lost tooth, she was enabled to take all her food and drink by using a tube which had to be well inserted into the mouth for that purpose. The ulceration included both cheeks, from the mouth upward to the orbits of the eyes, involving only the skin and subcellular tissue, but had destroyed the cartillages of the nose entirely. The lower eyelids were edamatous, and the conjunctivæ had a fiery appearance, and were at all times quite painful.

Her general health was not at all good. Once every month there was a regular return of intermittent fever, which, in the April after her admission, was accompanied with pelvic fulness and pains indicative of a menstrual effort; and this molumen returned with great regularity as long as she remained under my care, but without the menstrual flow accompanying it.

I found habitual constipation, and of course a very poor appetite. Her voice was harsh, as if the larynx was edamatous, and her thirst for cold water was incessant.

Such is a brief mention of the case as it presented itself to my observation, and for which, as it turned out, I was asked to exert, gratuitously, all the skill I possessed, in its cure. It was a gloomy outlook; the hopeful side of it being a probable recovery, with the inevitably repulsive face upon which the girl herself, as well as the world, would have to behold, with all the repugnance that such a disfigured picture would entail upon the feelings of the observers. On the other hand was death from a horrible standpoint, due to a disease almost regarded as an *opprobrium medicarum*, whilst the patient held her life in such high estimation as to plead piteously for the one chance out of a hundred, even if this disfiguration robbed her of all but the bare sympathies of a loveless solitude.

I began the treatment at once, by cleansing thoroughly the ulcerated surface, with a deoderizing wash of bromo-chloralum, ordering a tepid bath in which was diffused a supply of ley water for cleansing the entire surface, including the scalp, from which I clipped the beautiful suit of golden hair; and then covering the cancerous part with an unguent composed as follows, viz :

℞ Cera flava, ℥ij,

Spermacetti, ℥j,

Melt together and add,

Oil sweet almonds, }

Oil of turpentine, } *aa* ℥ss.

M. ft. unguentum. To be spread upon thin muslin and applied two or three times a day.

The application of this ointment was followed immediately by a decided improvement in the appearance of the ulcer, and in relieving the burning pains of the diseased surface. This was followed by two or three doses of mercurial aperients, which acted happily to the great relief of the digestive and nutritive functions; and then I put her upon what I had much reason to believe was perhaps the only remedy likely to subdue the violence of the corroding nature of the epitheliomatous eruption, —*arsenic*. Of this, four drops of Fowler's solution was given, in a ½ tumblerfull of water, just after each regular meal, prospectively to be continued for months, if necessary.

On the third day after this treatment was adopted, all the odor had disappeared, and for the suppuration but one application of the ointment daily was necessary. In fact, the healing process went on so vigorously that by the middle of April every part had healed beautifully, except a spot no larger than a dime, which was left on the face near the right angle of the mouth, and which lingered obstinately for several days, and then abhorently repullulated in spite of everything, until nearly the whole surface of the original seat of the cancer was again invaded. And although I was quite prepared to expect this explosion from former experiences, it was, nevertheless, very perplexing to my buoyant anticipations, for I had gotten to be almost of the mind of Socrates while dreamily dying from the effect of the hemlock, when he exclaimed to his friend who had, on account of his great love for the philosopher, insisted on remaining with him in prison until the death penalty had been executed: "Crito," said he, while drifting into death, and seeing the end near at hand, had just memory enough left him to say how surprised and glad he was to find his death so painless, "We owe a cock to Esculapius; discharge the debt, and by no means omit it." I, too, thought of the obligations we were under to the discoverer of the curative virtues of arsenic, and would willingly subscribe to the building of a magnificent temple to his memory, for the inestimable boon he had conveyed to suffering humanity.

Here, however, I had all my work to do over again; and, to

"make assurance doubly sure," I added to the treatment, as an application, a paste of iodide of arsenic, with just enough water to hold it in mass, and spread upon the ulcer. This was very painful for one hour, after which time the pain subsided never to return again, and twenty-four hours later the healing process began afresh, the restoration being almost as rapid as had been the respread of the ulceration.

Empirics get great credit for curing cases of chronic diseases with agents which they falsely claim as unknown to the profession, whilst in reality they are using well-known remedies, and deserve credit *only* for persevering industry with which they work, ignorant, and therefore unconscious of the danger which such agents might induce.

If there is one disease more than any other which requires constant and persistent energy in its management, that disease is, beyond doubt, the one we are now discussing; and it is a frequent reply with me, when asked for the explanation of my success in the treatment of epithelioma, "it is work, sir, work, and hard work at that."

This first repullulation occurred, as did all the subsequent backsets, just at the time of the menstrual effort, which was preceded by and accompanied with high fever of a quotidian type,, and, of course, all other treatment, except gentle applications to the ulcer, was temporarily omitted. As soon, however, as the exascertations of fever was controlled by quinia, the arsenic was again repeated, but in smaller doses, say three drops of the solution freely diluted with water, and, during the remaining existence of the disease, was continued intermittingly, for I had every assurance that the arsenical saturation of the system was complete, and that as the saturation declined, which is invariably the case in young subjects, it was only necessary to resort to the solution for three days, and omit it for the same length of time,to keep up the desired effect.

I will here digress a little from the task of reporting this case, and assert as a discovery of my own,from much experience with the medicine,that, in young subjects, arsenic may be pushed very freely without much apprehension of its toxicological effects, whilst in people above fifty years of age, the impressions made by this powerful agent, unless it is watched closely, will soon exhibit its virulence more or less permanently on the nervous, and end-osteal systems, by manifestations of pain, and disability of locomotion. This is an important matter to the physician, to the end that he can, by due vigilance be enabled to increase or diminish the amount of the medicine, or,as is my custom after indications of saturation are evident, to give for three days, and omit for the same length of time; because, on closely watching in all my cases, I have found that the effects of arsenic on the human system are full in three days administration, and begin to decline after the third day of omission.

But to return from this impressive digression, which I conceived to be of too important a nature to pass without an appropriate notice, I will resume the original thread of my subject.

Within ten days after the regular treatment was resumed, the ulceration had again healed to a small place no larger than two lines in dimensions, and the little patient was gaining rapidly in flesh and spirits, which continued steadily until the middle of May; at which time there was another effort at repullulation, but much less marked than before. By the middle of June the parts had healed up to a very small point on the opposite cheek, and again began to spread, but very feebly. At the next molimen menstruale, the face was healed over all but a small pimple-like spot in the same place as last mentioned. The fever had also become less severe, and more easily under control of quinia. And by the first of August the recovery was as perfect as my most sanguine wishes could have anticipated. At this time I began giving her tincture of chloride of iron, in twenty drop doses, three times a day continuously, with the Fowler's solution at such times as she had to take that medicine, but omitted at no time for the next two months,under the hope that, by its use, nature would be assisted in the elimination of the menstrual flow, which I had every reason to believe could not be many periods longer delayed. But up to the time of her departure in November, for her home in Louisiana, this hope was not realized.

I heard frequently from her afterwards, and every report assured me that her recovery from her terrible affliction was as perfect as could be wished; and in passing her residence on the first day of January, 1880, I had a momentary inspectation of her face, which had the appearance of being in good condition, and perfectly recovered.

There is but little question now amongst medical scientists, that lupus exedens is a true epithelioma; and in Gross' Surgery the author asserts unequivocally that the two apparent diseases are really typical instances of an epitheliomatous nature ; or, in other and better language,lupus exedens is typical epithelioma.

The first case of this disease which came under my treatment was a perfect specimen of the olden time *noli me tangere*, seated in the septum of the nose, and although its progress was cut short in ten days, it left quite a chasm permanently as an evidence of its violent character. At that time Dr. J. C. Simons, of this city, then a resident of Greensboro, Alabama, was associated with me in its treatment, and, through him, I became familiarly acquainted with the caustic influence of the muriate of antimony, ordinarily called butter of antimony, upon which we relied as a local application in the case, and with the happiest results, when used in connection with Fowlers' Solution internally, which I was then administering to the lady patient in ten drop doses three times a day, but who had taken hardly half an ounce of the medicine before swelling and pains in the feet set in; and although this lady lived twenty years longer, she complained,to the last,of her feet, and inability of walking with steadiness. She was above fifty years of age at the time of the attack of the *noli me tangere*, from which age I had subsequent reason to infer that the arsenic had been the cause of the local distress. Afterwards I treated quite a number of elderly patients for different forms of epithelioma of the face, and although I had become somewhat more cautious in the use of arsenic, now and then a case would go hoblingly along from its toxicological effects the rest of their lives ; but in no case was there ever a return of the disease for which they were treated. In one case, I remember, where the disease was ulcerating at the inner canthus of the right eye of an old man, the internal rectus muscle was so deeply involved in the disease as to permit the globe of the eye to turn helplessly to the outer angle of the orbit, so that the pupil was completely hidden away, but when the disease had perfectly recovered, the muscle regained its controlling power, and the functions of the eye became restored without any additional assistance on my part.

Since my removal to this city, I have seen only three cases of epithelioma, two of them being situated on the cheeks of men of 65 and 70 years of age respectively, and who had been afflicted with intestinal dyspepsia for several years before the outbreak of the cancerous trouble. To these patients I could offer no hope of cure, unless digestion and nutrition could be,in the first place,re-established. ; but in the meantime, I filled the cancerous cavities with saccharated pepsine, which, in a short time, removed the sloughy coating, arrested the profuse suppuration, and established healthy granulations in a very hopeful manner. I cannot too highly commend to your attention the use of pepsine in chronic ulcerations, which when aided by the arsenic internally,will, I believe, invariably re-establish healthy granulations, irrespective of the character of the disease that produced the forbidding sulcus.

The third case was a boy of 12 years of age, thin, sallow,and morose from malarial infection, and upon whose nose appeared at last a warty excressence which continued growing, with a larger and larger bleeding surface, until the parents became frightened,and brought him to Dr. Alexander, of this city, for treatment. The Doctor requested my association with him in the case, and under the influence of iron and arsenic,and iodide of arsenic,· as an application to the bleeding tumor, the lad rapidly regained a healthful appearance, and the excressence was, in one month,healed so perfectly as to leave hardly a scar on the spot where the tumor was situated.

Upon looking over my experience in this department of the surgical field, I am as pleasurably refreshed with the success of arsenic and its various salts, in the cure of epithelioma, as was said to be the devotees of Mahomet, whilst quaffing the water in which the body of the prophet had just been bathed. For, out of fifty-two cases placed under treatment with the medicine, there was not a single failure to record, and many of the patients are still living as monuments of the effective agency of this inestimable boon.

